# Supplementary material for: Venomix: a simple bioinformatic pipeline for identifying and characterizing toxin gene candidates from transcriptomic data
Source: PeerJ. 2018 Jul 31;6:e5361. doi: 10.7717/peerj.5361 (PMC6074769; doi:10.7717/peerj.5361)
Supplement: Supplemental Information 3 [file peerj-06-5361-s003.gz › FinalOutput_E-6/Zinc_metalloproteinase-disintegrin-like_mikarin_1/finaltree.pdf]

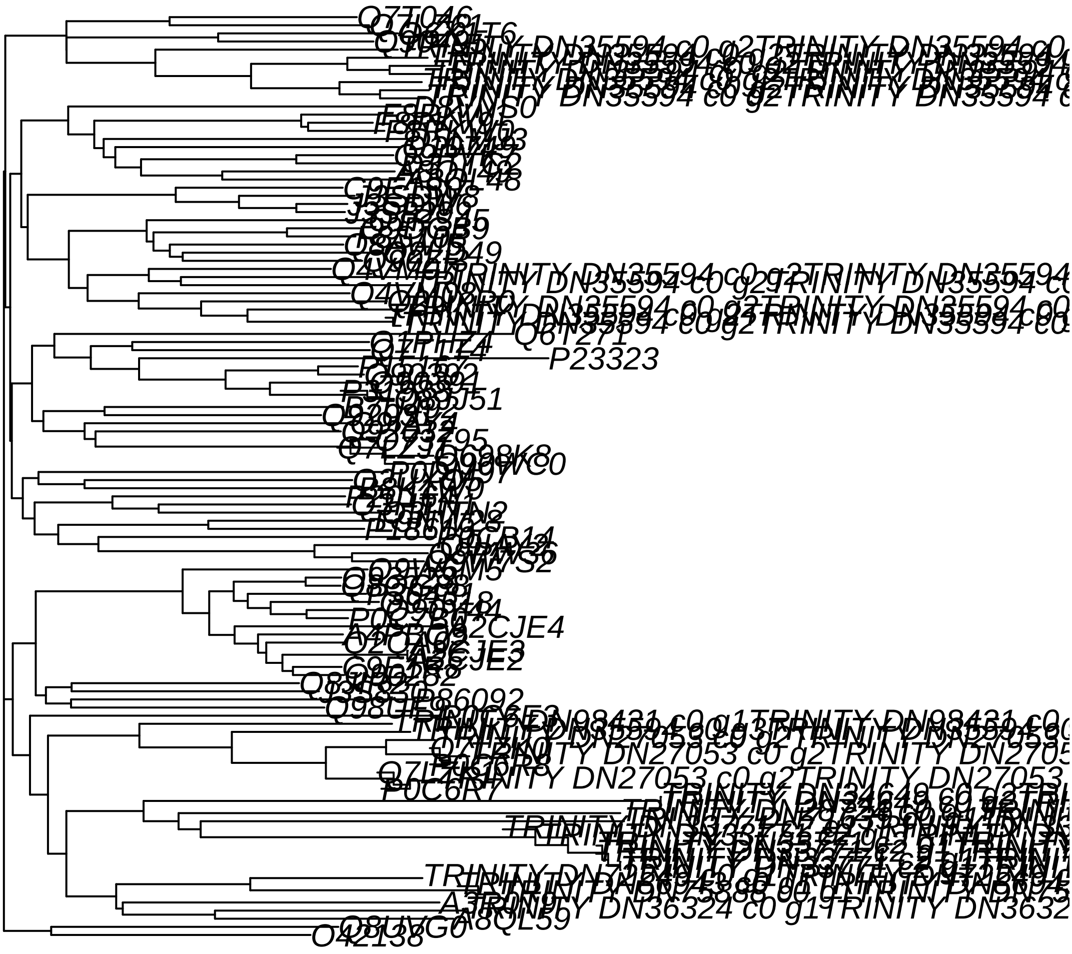
Q7T046
P23323
Q6T271
TRINITY DN27053 c0 g2TRINITY DN27053
P0C6R7
Q8UVG0
O42138
